# Prevalence and determinants of non-communicable disease risk factors among adult population of Kathmandu

**DOI:** 10.1371/journal.pone.0257037

**Published:** 2021-09-08

**Authors:** Sitasnu Dahal, Ram Bilakshan Sah, Surya Raj Niraula, Rajendra Karkee, Avaniendra Chakravartty

**Affiliations:** 1 School of Public Health and Community Medicine, B.P. Koirala Institute of Health Sciences, Dharan, Nepal; 2 Faculty of School of Public Health and Community Medicine, B.P. Koirala Institute of Health Sciences, Dharan, Nepal; University Lyon 1 Faculty of Dental Medicine, FRANCE

## Abstract

**Background:**

According to WHO, the deaths due to NCDs in Nepal have soared from 60% of all deaths in 2014 to 66% in 2018. The study assessed the prevalence and determinants of non-communicable disease risk factors among adult population of Kathmandu.

**Materials and methods:**

A community based cross-sectional study was conducted from September 2019 to February 2020 among 18–69 years adults residing in municipalities of Kathmandu district. Multi-stage random sampling technique was used to select 245 subjects who were interviewed using WHO NCD STEPS instrument. Chi-square test and logistic regression analysis were done to explore the determinants of NCD risk factors.

**Results:**

The prevalence of current smoking, alcohol consumption, low intake of fruits and vegetables and low physical activity was found to be 22%, 31%, 93.9% and 10.2% respectively. More than half (52.2%) of the participants were overweight or obese and the prevalence of raised blood pressure was 27.8%. Smoking was associated significantly with male gender (AOR = 2.37, CI: 1.20–5.13) and respondents with no formal schooling (AOR: 4.33, CI: 1.50–12.48). Similarly, the odds of alcohol consumption were higher among male gender (AOR: 2.78, CI: 1.47–5.26), people who were employed (AOR: 2.30, CI: 1.13–4.82), and those who belonged to Chhetri (AOR: 2.83, CI: 1.19–6.72), Janajati (AOR: 6.18, CI: 2.74–13.90), Dalit and Madhesi, (AOR: 7.51, CI: 2.13–26.35) ethnic groups. Furthermore, respondents who were aged 30–44 years (AOR: 5.15, CI: 1.91–13.85) and 45–59 years (AOR: 4.54 CI: 1.63–12.66), who were in marital union (AOR: 3.39, CI: 1.25–9.13), and who belonged to Janajati (AOR: 3.37, CI: 1.61–7.04), Dalit and Madhesi (AOR: 4.62, CI: 1.26–16.86) ethnic groups were more likely to be associated with overweight or obesity. Additionally, the odds of raised blood pressure were higher among people who were of older age (AOR: 6.91, CI: 1.67–28.63) and those who belonged to Janajati ethnic group (AOR: 3.60, CI: 1.46–8.87) after multivariate analysis.

**Conclusion:**

The findings of the study highlighted high prevalence of behavioral and metabolic risk factors, which varied on different socio-demographic grounds. Thus, population specific health promotion interventions centered on public health interests is recommended to reduce risk factors of NCDs.

## Introduction

Non-communicable diseases (NCDs) have garnered a global attention as it afflicts a wide range of population both in developed and in developing countries. Globally, NCDs kill 41 million people each year, equivalent to 71% of all deaths and 85% of premature deaths, aged 30 and 69 years; occur in low and middle-income countries [1]. The grappling effect of NCDs in low and middle-income countries (LMICs) surprises those who think maternal and child deaths, deaths due to infectious diseases are only the dominant causes of mortality [2]. NCDs are top killers in South East Asia Region, claiming an estimated 8.5 million lives each year, of which one-third are premature, thus affecting labor supply and economic productivity [3].

The World Health Organization (WHO) has focused on four NCDs that are driving the epidemiological transition: diabetes, cardiovascular disease, chronic respiratory disease, and cancers and they are largely caused by behavioral and metabolic risk factors [1]. Modifiable behavioral risk factors include tobacco use, harmful use of alcohol, unhealthy diet and physical inactivity whereas metabolic risk factors include raised blood pressure, overweight/obesity, hyperglycemia and hyperlipidemia [1]. Kishore et al. posit, “these risks are largely man-made and relate to how we live, age, work and play and are driven by urbanization, population ageing and trade such that premature death and disability due to NCDs can therefore be viewed as failures of a broader socioeconomic system” [4].

A report on Global Health Risks by World Health Organization mentioned that, in terms of attributable deaths, the leading NCD risk factor globally is raised blood pressure (to which 13% of the global deaths are attributed), followed by tobacco use (9%), raised blood glucose (6%), physical inactivity (6%) and overweight and obesity (5%) [5]. In Nepal, NCDs are estimated to account for 66% (2016) of all deaths [6]. The nationwide STEPS (STEPwise approach for Surveillance) survey of non-communicable disease risk factors carried out in 2019 showed a high prevalence of less than five servings of fruit and/or vegetable (96.7%), tobacco use (28.9%), overweight/obese (24.3%), raised blood pressure (24.5%), posing a greater threat for non-communicable diseases epidemic in future [7]. Several studies conducted have concluded that NCDs risk factors vary by socio-demographic characteristics [8–12].

Kathmandu, being biggest urban hub in Nepal, is experiencing rapid nutritional transition, lifestyle changes, and epidemiological transition following modernization, westernization and increased reliance in technology. A decrease in single risk factor can save a person from multiple NCDs. The strong association between NCDs and the identified risk factors has highlighted the need to measure trends of NCDs risk factors and its variation in regard to socio-demographic characteristics to emphasize the need and progress of preventive activities. Therefore, the study aims to assess the distribution and correlates of NCDs risk factors; specifically, the prevalence and determinants of behavioral (smoking, alcohol consumption, unhealthy diet and low physical activity) and metabolic (overweight/obesity and raised blood pressure) risk factors of selected NCDs among adult population of Kathmandu.

## Methods

### Study design and setting

This was a community based, cross-sectional study carried out in the municipalities of Kathmandu district from September 2019 to February 2020. Study site represents most densely populated district of Nepal; Kathmandu where haphazard and unplanned urbanization is evident posing multi-hazard risk to the population [13].

### Study population, sample size and sampling technique

Study population included adult men and women of 18–69 years who were residing in Kathmandu district for at least six months. However, pregnant women, those who were too frail and mentally unfit, those with physical disability and those not consenting for the study were excluded.

Sample size was calculated using the formula for estimating one sample proportion (n = Z^2^pq/l^2^) with specified relative precision, where the estimated prevalence of hypertension was 34.4% [10]; allowable error 20%; level of significance 5%, and non-response rate 20%, which gave out a sample size of 222. However, 245 samples were enrolled in the study.

A multistage random sampling technique was employed to select 245 participants. Kathmandu district was purposively selected. Of the 10 municipalities in Kathmandu, two municipalities; Kageshwori Manohara and Budhanikantha were randomly selected using simple random technique. Again, two wards from each municipality were selected using simple random technique (lottery method). The sample size was then equally allocated to each ward. A systematic random sampling method was then applied to choose 245 households. If more than one eligible respondent were present, one individual from that household was enrolled in the study through simple random technique (lottery method). If eligible respondent was not available, participant was selected from adjacent household.

### Data collection tool and technique

Data was collected using WHO STEPS questionnaire [14] to collect information on behavioral and metabolic risk factors. Face-to-face interview was conducted, and anthropometric and clinical measurements were taken. The questionnaire was pre-tested (10% of sample size) in similar setting and the investigator herself did the data collection.

#### Tobacco use

Data particularly related to the pattern of smoked and smokeless tobacco products including the age of initiation, frequency of use and exposure to second-hand smoking were collected from tobacco users. Current smoking were those who had smoked tobacco products in the last 30 days [14].

#### Alcohol consumption

Data pertaining to lifetime abstinence, alcohol consumption in the past 12 months, and heavy episodic drinking was obtained from the respondents. Pictorial show cards featuring different kinds of glasses and bowls were shown to help them recall the amount of drinking. The self-reported amount was then used to determine the number of standard drink of alcohol consumed (one standard drink is equal to 10 g of ethanol) [14]. Current episodic heavy drinking was considered as six or more drinks on any day in the past 30 days [14].

#### Diet

Dietary recall method was used to record the number of days of consumption of fruit and/or vegetables in a typical week and the number of servings of fruit and/or vegetables consumed in an average in a day. Less than five servings of fruits and/or vegetables intake per day was considered insufficient [14]. Information regarding types of cooking oil used and amount of salt consumed by a family in a month was also collected.

#### Physical activity

Physical activity was analyzed using the Global Physical Activity Questionnaire (GPAQ) and total physical activity <600 MET (Metabolic Equivalent of Tasks) minutes per week was categorized as low physical activity [14, 15].

#### Anthropometric measurement

Height was measured in centimeter with a portable standard stature scale without foot and headwear. Weight was recorded in kilograms with a portable digital scale by placing on a firm, flat surface. A body mass index (BMI) of ≥ 30.0 and 25.0–29.9 kg/m^2^ was considered obese and overweight respectively.

Waist and hip circumferences were measured by a constant tension tape following STEPS Manual guideline [14]. Based on WHO criteria, waist hip ratio scores of ≥0.90 cm for male; and ≥0.85 cm for female were considered as substantially increased [16].

#### Clinical examination

Blood pressure was measured using Doctor’s Aneroid Sphygmomanometer with an appropriate sized cuff. Raised blood pressure was defined as having systolic blood pressure ≥140 mm of Hg and/or diastolic blood pressure ≥90 mm of Hg during the study, or being previously diagnosed as having hypertension. This was determined by documentation such as a treatment record book, or participant history of medication for high blood pressure [14].

### Data management and analysis

Data were compiled, edited, and checked for consistency, and processed through Microsoft Excel, 2011 and SPSS V.21.0 for further analysis. Frequency and percentage were presented in tables and charts to show the distribution of risk factors among socio-demographic characteristics. Bivariate analysis was done using Chi-square test to find out the significant association between NCDs risk factors and other selected variables at 95% confidence interval. Explanatory variables showing p value of less than 0.2 in bivariate analysis were entered into the logistic model for multivariate analysis to calculate adjusted odds ratio and p-value less than 0.05 was considered statistically significant.

### Ethical consideration

The study protocol was approved by Institutional Review Committee of BP Koirala Institute of Health Sciences (BPKIHS) and Ethical Review Board (ERB) of Nepal Health Research Council (NHRC). The purpose of the study and procedures were explained and written informed consent was gained before commencing the data collection. The participants were also informed that their participation will be voluntary and can withdraw at any moment. Moreover, they were assured regarding their anonymity, and the confidential treatment of their responses.

## Results

### Demographics

The mean ages for males and females were 41.19±14.49 and 40.91±12.67 years respectively. More than half of the participants (52.7%) were female. The majority of the respondents (35.9%) belonged to age group 30–44 and most of them were from Brahmin (33.9%) and Janajati (33.5%) ethnic groups (Table 1). More than eighty percent were in marital union and more than forty percent had higher education (Table 1). Nearly two-third of the respondents were working and 71.8% of the respondents were above poverty line (≥1.9$/head/day) (Table 1). The median family income/month and inter-quartile range (IQR) was NRs 40,000 (10,000–9,00,000).

**Table 1:** Distribution of socio-demographic characteristics by gender (n = 245).

Socio-demographic characteristics		Gender		Total No. (%)	P Value
		Male No. (%)	Female No. (%)		
**Age group (in years)**	18–29	29 (25.0)	28 (21.7)	57 (23.3)	0.985
	30–44	41 (35.3)	47 (36.5)	88 (35.9)	
	45–59	30 (25.9)	43 (33.3)	73 (29.8)	
	60–69	16 (13.8)	11 (8.5)	27 (11.0)	
**Caste/Ethnic groups**	Brahmin	39 (33.6)	44 (34.1)	83 (33.9)	0.860
	Chhetri	31 (26.7)	33 (25.6)	64 (26.1)	
	Janajati	37 (31.9)	45 (34.9)	82 (33.5)	
	Dalit and Madhesi	9 (7.8)	7 (5.4)	16 (6.5)	
**Marital Status**	Not in union	27 (23.3)	21 (16.3)	48 (19.6)	0.169
	Union	89 (76.7)	108 (83.7)	197 (80.4)	
**Education Level**	No formal education	12 (10.4)	41 (31.8)	53 (21.6)	<0.001*
	Primary	28 (24.1)	33 (25.6)	61 (24.9)	
	Secondary	21 (18.1)	11 (8.5)	32 (13.1)	
	Higher education	55 (47.4)	44 (34.1)	99 (40.4)	
**Work Status**	Working	94 (81.0)	64 (49.6)	158 (64.5)	<0.001*
	Not working	22 (19.0)	65 (50.4)	87 (35.5)	
**Income**	Above poverty line	85 (73.3)	91 (70.5)	176 (71.8)	0.671
	Below poverty line	31 (26.7)	38 (29.5)	69 (28.2)	

There was no statistical difference in the demographics of the participants by gender, however, significant statistical difference were found in education level and working status group by gender; more males had higher education as compared to females and more females were not working as compared to males (Table 1).

### Prevalence and determinants of NCDs risk factors

#### Smoking

The overall prevalence of current smoking was 22.0% (Table 2). The mean years of initiation of smoking±SD were 17.13±6.74 and the median number of sticks smoked per day (IQR) was 5(3–10). Out of the total respondents, one–fifth of them used smokeless tobacco and 43.7% and 48.6% of the respondents were exposed to second-hand smoke at home and at workplace respectively (Table 2). It was found that males were more likely to be associated with smoking in comparison to females (AOR = 2.37, CI: 1.20–5.13) (Tables 3 and 4). Respondents having no formal schooling were 4.33 times more likely to smoke in comparison to those who had higher education (CI: 1.50–12.48) after multivariate analysis (Table 4).

**Table 2:** Prevalence of NCD risk factors among adult population of Kathmandu n = 245.

Variables	Characteristics	Category	N (%)
**Behavioral risk factors for NCDs**			
**Smoking**	Smoking (current)	Yes	54 (22)
		No	191 (78)
	Smoking (daily)	Yes	46 (18.8)
		No	199 (81.2)
	Smokeless tobacco (current)	Yes	49 (20)
		No	196 (80)
	Smokeless tobacco (daily)	Yes	45 (18.4)
		No	200 (81.6)
	Passive smoking at home	Yes	107 (43.7)
		No	138 (56.3)
	Passive smoking at workplace	Yes	119 (48.6)
		No	126 (51.4)
**Alcohol Consumption**	Lifetime abstainers	Yes	165 (67.3)
		No	80 (32.7)
	Alcohol consumption in 12 months	Yes	76 (31.0)
		No	169 (69.0)
	Alcohol consumption in 30 months	Yes	74 (30.2)
		No	171 (69.8)
	Heavy Episodic Drinking	Yes	31 (12.7)
		No	214 (87.3)
**Fruits and/or vegetable intake**	No. of servings	< five servings per day	230 (93.9)
		≥ five servings per day	15 (6.1)
**Physical activity**	Level of physical activity	Sufficient (≥ 600 MET minute/week)	220 (89.8)
		Insufficient (<600 MET minute/week)	25 (10.2)
**Salt Consumption**	Amount	≤ 5g per day	18 (7.3)
		> 5g per day	227 (92.7)
**Metabolic risk factors for NCDs**			
**Overweight/obese**	> 24.9 kg/m^2^	Yes	128 (52.2)
		No	117 (47.8)
**Raised blood pressure**	≥140 mm of Hg systolic and/or ≥ 90 mm of Hg diastolic or previously diagnosed hypertension	Yes	68 (27.8)
		No	177 (72.2)

**Table 3:** Association of NCD risk factors by socio-demographic characteristics (n = 245).

Variables	Categories	Smoking (%)	Alcohol Consumption (%)	Insufficient fruits and vegetable intake (%)	Insufficient physical activity (%)	Overweight or obese (%)	Raised blood pressure (%)
**Age (in years)**	18–29	10.5	24.6	93.0	8.8	21.1	12.3
	30–44	23.9	39.8	97.7	11.4	67.0	25.0
	45–59	21.9	28.8	87.7	11.0	60.3	35.6
	60–69	40.7	22.2	100	7.4	48.1	48.1
	P–value	0.007*	0.692	a	a	0.004*	<0.001*
**Gender**	Male	26.7	43.1	96.6	7.8	48.3	31.0
	Female	17.8	20.2	91.5	12.4	55.8	24.8
	P–value	0.094	<0.001*	0.098	0.230	0.238	0.277
**Marital status**	Not in union	10.4	29.2	97.9	8.3	18.8	14.6
	Union	24.9	31.5	92.9	10.7	60.4	31.0
	P–value	0.03*	0.757	0.315^b^	0.633^b^	<0.001*	0.023*
**Ethnicity**	Brahmin	16.9	13.3	90.4	9.6	37.3	20.5
	Chhetri	23.4	29.7	95.3	9.4	51.6	18.8
	Janajati	23.2	46.3	95.1	9.8	65.9	41.5
	Dalit and Madhesi	37.5	50.0	100.0	18.8	62.5	31.3
	P–value	0.103	<0.001*	a	a	<0.001*	0.006*
**Education level**	No formal education	35.8	22.6	90.6	9.4	56.6	32.1
	Primary	29.5	41.0	95.1	11.5	70.5	29.5
	Secondary	15.6	31.3	93.8	6.3	43.8	37.5
	Higher education	12.1	29.3	94.9	11.1	41.4	21.2
	P–value	<0.001*	0.900	a	a	0.005*	0.150
**Work status**	Working	24.7	38.6	95.6	9.5	58.2	31.6
	Not working	17.2	17.2	90.8	11.5	41.4	20.7
	P–value	0.179	<0.001*	0.166^b^	0.621	0.012*	0.067
**Income**	Above poverty line	19.3	28.4	93.8	8.5	52.3	30.1
	Below poverty line	29.0	37.7	94.2	14.5	52.2	21.7
	P–value	0.101	0.158	1.000^b^	0.165	0.989	0.188

*: Significant at P<0.05, a: Expected cell count <5, b: Fischer Exact Test.

**Table 4:** Multivariate logistic regression to determine predictors of NCD risk factors (n = 245).

Characteristics	Smoking (%)	Alcohol Consumption (%)	Overweight or obese (%)	Raised blood pressure (%)
	Hoshmer and Lemeshow Test = 0.21	Hoshmer and Lemeshow Test = 0.74	Hoshmer and Lemeshow Test = 0.20	Hoshmer and Lemeshow Test = 0.77
**Age group (in years)**				
18–29	Ref	Ref	Ref	Ref
30–44	1.66 (0.49–5.61)	a	5.15 (1.91–13.85)*	1.98 (0.65–5.97)
45–59	1.21 (0.34–4.37)	a	4.54 (1.63–12.66)*	3.35 (1.14–9.82)*
60–69	2.50 (0.61–10.23)	a	2.88 (0.85–9.72)	6.82 (1.95–23.79)*
**Gender**				
Female	Ref	Ref	Ref	Ref
Male	2.37 (1.20–5.13)*	2.78 (1.47–5.26)*	a	A
**Marital status**				
Not in union	Ref	Ref	Ref	Ref
Union	1.50 (0.44–5.10)	a	3.29 (1.25–8.66)*	1.56 (0.54–4.48)
**Ethnicity**				
Brahmin	Ref	Ref	Ref	Ref
Chhetri	1.52 (0.63–3.67)	2.83 (1.19–6.72)*	1.71 (0.83–3.54)	0.89 (0.38–2.08)
Janajati	1.13 (0.48–2.65)	6.18 (2.74–13.90)*	3.37 (1.61–7.04)*	3.07 (1.48–6.36)*
Dalit and Madhesi	2.69 (0.73–9.92)	7.51 (2.13–26.35)*	4.62 (1.26–16.86)*	2.45 (0.70–8.59)
**Education level**				
Higher education	Ref	Ref	Ref	Ref
No formal education	4.33 (1.50–12.48)*	a	0.71 (0.29–1.70)	A
Primary	2.31 (0.89–6.0)	a	1.20 (0.53–2.69)	A
Secondary	0.93 (0.27–3.21)	a	0.53 (0.21–1.36)	A
**Work status**				
Not working	Ref	Ref	Ref	Ref
Working	1.22 (0.53–2.82)	2.30 (1.12–4.73)*	1.05 (0.54–2.01)	A
**Income**				
Above poverty line	Ref	Ref	Ref	Ref
Below poverty line	1.46 (0.72–2.97)	1.73 (0.89–3.38)	a	a

Ref: Reference group,

*significant at p<0.05, a = not significant in bivariate analysis then dropped.

#### Alcohol consumption

Of the total respondents, more than two–third (67.3%) were lifetime abstainers. The overall prevalence of alcohol consumption in last 12 months was 31.0% and 12.7% of the respondents were involved in heavy episodic drinking (Table 2). It was observed that males were 2.78 times more likely to consume alcohol than females (CI: 1.47–5.26). It was also evident that ethnic groups, Chhetri (AOR: 2.83, CI: 1.19–6.72), Janajati (AOR: 6.18, CI: 2.74–13.90), Dalit and Madhesi, (AOR: 7.51, CI: 2.13–26.35) were more likely to consume alcohol than Brahmin. Likewise, respondents who were working were 2.30 times more likely to drink alcohol than who were not working (CI: 1.13–4.82) (Table 4).

#### Fruits and/or vegetable consumption

The study showed that 93.9% of the respondents consumed less than 5 servings of fruits and/or vegetable. Only 6.1% of the respondents consumed recommended five or more servings of fruits and vegetables per day (Table 2). It was observed that none of the socio-demographic factors were statistically significant with dietary serving of fruits and vegetable (Table 3)

#### Physical activity

The prevalence of low physical activity (<600 METs minutes/week) in form of work, travel to and from places, recreational activities, was found to be 10.2% (Table 2). None of the study variables were statistically significant with physical activity.

#### Salt consumption

More than ninety percent of the sampled population consumed >5g of salt per day (Table 2). More than half of the participants (56.7%) opined that high salt intake causes health problem and majority of the respondents (70.2%) used refined vegetable oil (mustard, soybean, sunflower oil) for cooking.

#### Overweight and/or obesity

**Fig 1:**
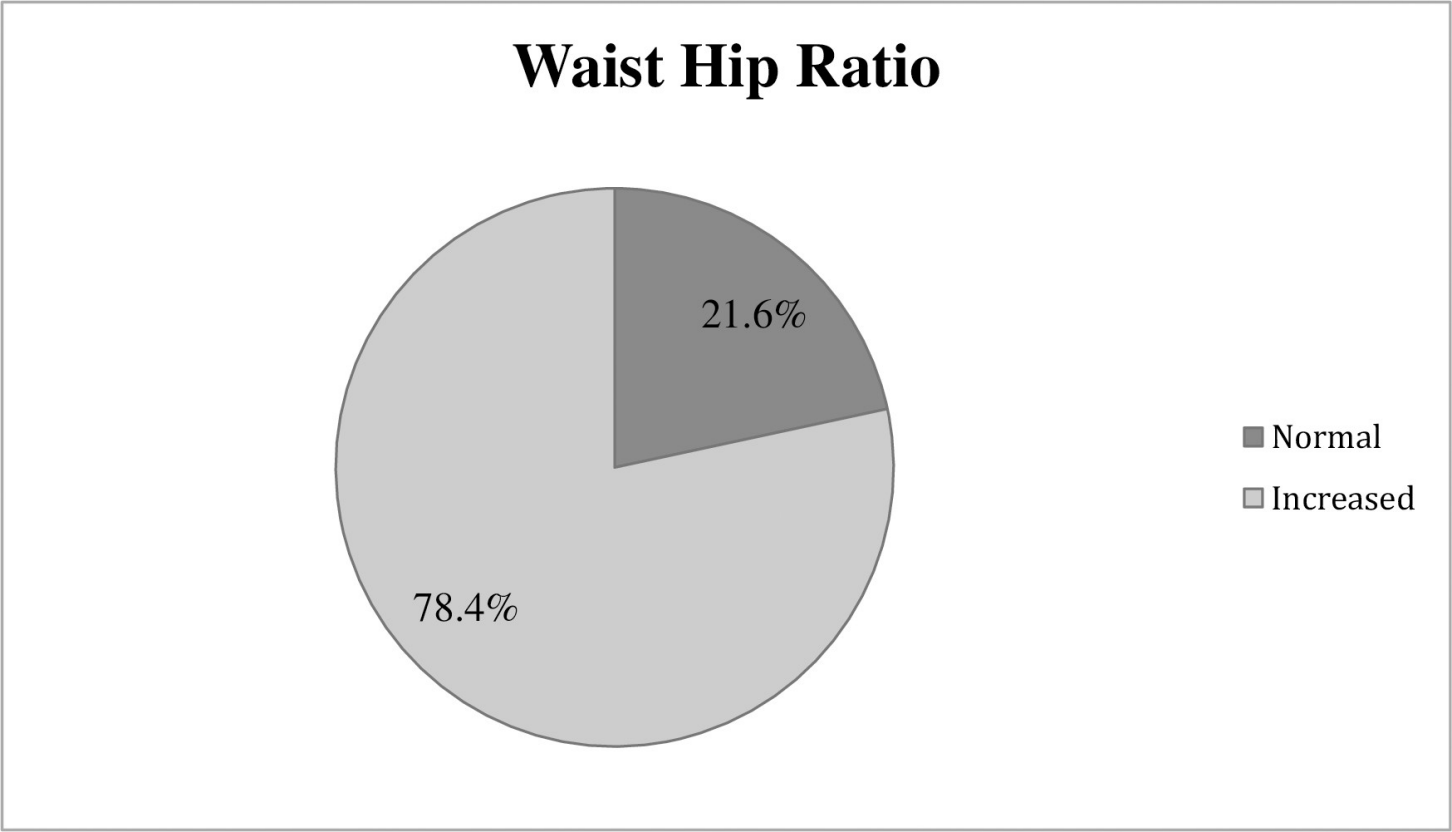
Waist-hip ratio among the adult population of Kathmandu (n = 245).

The mean body mass index of the participants and standard deviation were (25.42±4.56) kg/m^2^. The overall prevalence of overweight/obesity was 52.2% of the total population (Table 2) and a higher prevalence was seen in females than in males (Table 3). More than three–fourth of the respondents had waist hip ratio substantially increased (Fig 1). The odds of being overweight and/or obese was higher in age groups 30–44 years (AOR: 5.15, CI: 1.91–13.85) and 45–59 years (AOR: 4.54 CI: 1.63–12.66), respondents who were in union (AOR: 3.29, CI: 1.25–8.66), and those who belonged to Janajati (AOR: 3.37, CI: 1.61–7.04), Dalit and Madhesi ethnic groups (AOR: 4.62, CI: 1.26–16.86) (Table 4).

#### Raised blood pressure

The prevalence of raised blood pressure, including those who were on medication for hypertension was 27.8% (Table 2). Higher (31.0%) prevalence was observed among males than in females (Table 3). Additionally, only 57.1% of the respondents who were hypertensive were under medication. It was observed that the participants of age group 45–59 years and 60–69 years were more likely to have raised blood pressure than participants of age group 18–29 years, which was statistically significant. Similarly, respondents who were from Janajati ethnic group were more likely to have raised blood pressure than respondents who were from Brahmin ethnic group, which was statistically significant (AOR: 3.07, CI: 1.48–6.36) (Table 4).

### Clustering of risk factors

**Fig 2:**
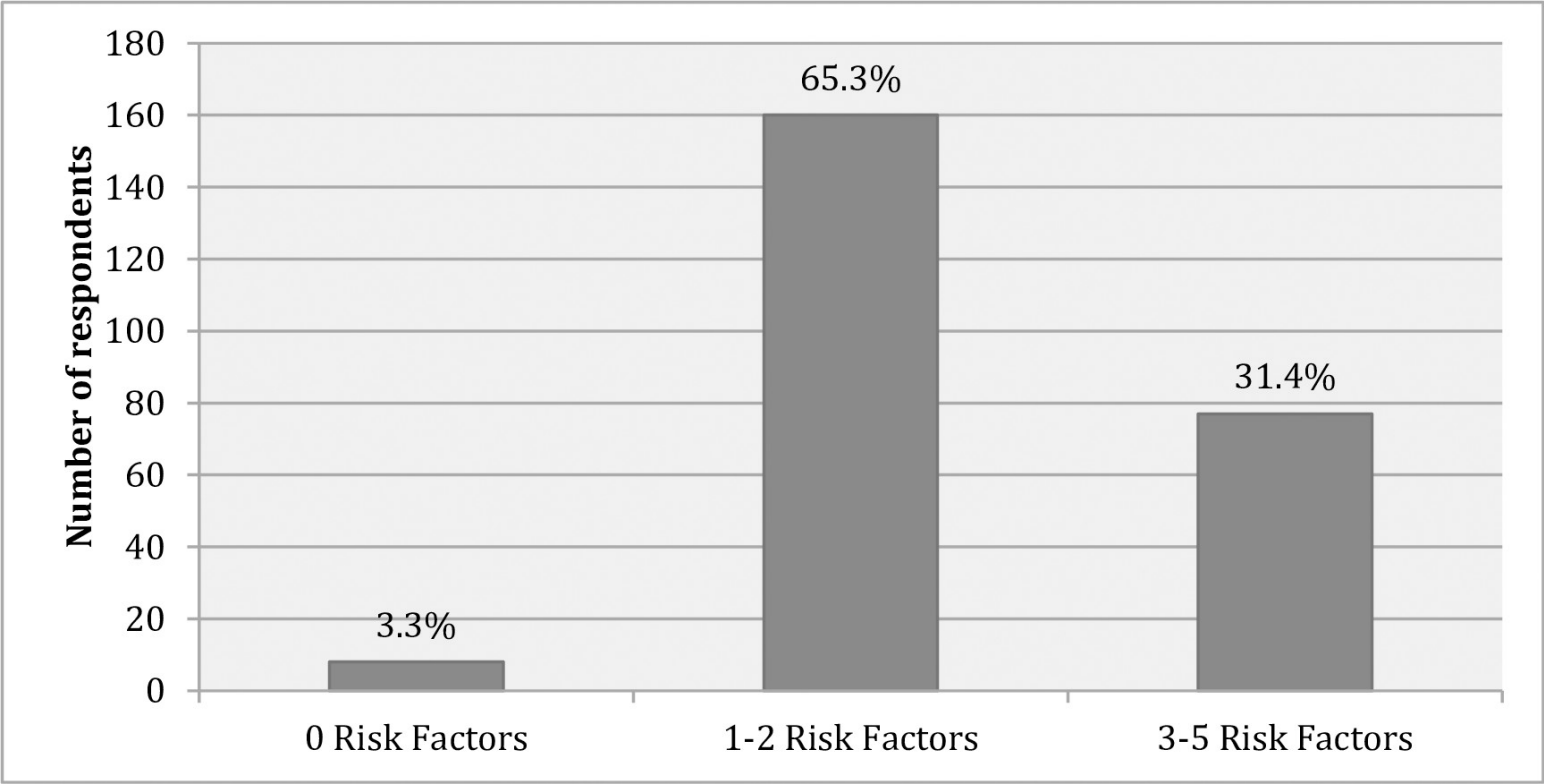
Combined risk factors among the adult population in Kathmandu (n = 245).

Combined risk factors included current daily smoking, less than 5 servings of fruits and/or vegetables, low physical activity, overweight and obese, and raised blood pressure [14]. Nearly two–third (65.3%) of the participants had 1–2 risk factors followed by 31.4% of the respondents with 3–5 risk factors and only 3.3% of the participants were with no risk factors (Fig 2).

## Discussion

This cross-sectional study assessed the prevalence and determinants of major non-communicable disease risk factors in adult population of Kathmandu–both modifiable behavioral risk factors (current smoking, alcohol consumption, low fruits and vegetable intake, physical inactivity) and biological/metabolic risk factors (overweight and/or obesity and raised blood pressure). The study has demonstrated high prevalence of certain NCD risk factors, such as current smoking, alcohol consumption, low fruits and vegetable consumption, and overweight/obesity. In addition, there is a wide variation in prevalence of risk factors by socio-demographic characteristics.

### Behavioral risk factors

The prevalence of current smoking is 22%, which is consistent with the 2013 NCD risk factors survey of Nepal (19%) [8]. The prevalence is high in comparison to studies conducted in peri-urban community of Kathmandu (17.6%) [10], South Africa (13.7%) [9], and Bolivia (11.6%) [17]. However, it is lower than the studies done in slum area of Kathmandu, Nepal (35.6%) [18], Bhutan (24.8%) [19], Darjeeling, India (69.8%) [20], and Pakistan (48.2%) [21]. This highlights the expedient need to control smoking and tobacco use as such activities are extremely injurious to human health. The present study also showed that smoking was associated with gender and education. The findings concur with other studies conducted in Nepal [8], Saudi Arabia [22]. The perception of women smoking as a social taboo might have made females more restricted to smoking than men. Since gender and education are strong predictors of smoking, comprehensive tobacco control law should reach among poorly educated people and male gender, which will aid in reducing the gaps and prevent people from adverse effects of smoking.

The prevalence of alcohol consumption (in last 12 months) is 31%, which is consistent in comparison to national data of 2019 Province 3 NCD risk factors survey (33.2%) [23] but high in comparison to the study conducted in South Africa (16.3%) [17]. The prevalence of heavy episodic drinking is 12.7% among total sampled population, which is higher than the 2019 NCD risk factors survey (6.8%) [7]. As the study has considered urban clusters where the liquor shops are easily accessible and social gatherings are incomplete without drinking, therefore, higher prevalence might have been reported. The study findings showed a strong association between alcohol consumption and gender, ethnicity and occupation. More males were found to consume alcohol than female, which concurs with the studies, conducted in Delhi, India [24], South Africa [9], 2013 nationwide STEPS Survey of Nepal [8]. Gender differences in alcohol consumption remain universal. Also the prevailing societal norm, which considers male drinking as normal behavior and female drinking as an immoral act may be one of the possible explanation. However, an increasing trend of female consumption of alcohol is seen in comparison to 2019 STEPS survey factsheet which is more an urban phenomenon [7]. Likewise, Chhetri, Janajati, Dalit and Madhesi ethnicity were found to consume more alcohol than Brahmins. A similar result is reflected in the study conducted in western Nepal where Dalit and Janajati were found to consume more alcohol [25]. Alcohol consumption is usually restricted by upper-castes such as Brahmins, while some ethnic groups in Nepal regard alcohol as pure offerings to God with major religious significance. Also the study has found occupation as a predictor of alcohol consumption. Employed respondents were two times more likely to consume alcohol than unemployed respondents. The result is inconsistent with the findings of meta-analyses and reviews of cross-national studies [26] which posit that adult unemployment is associated with increased levels of alcohol use. The rising cost of alcoholic beverages and as social gatherings are incomplete without alcohol nowadays might be the possible explanations for the result.

The prevalence of low fruits and vegetable intake is 93.9%, which is consistent with the findings of South Africa (93.5%) [9]. It is lower than the study conducted in North India (99.2%) [27], 2019 NCD risk factors survey (96.7%) [7]. However, it is higher in comparison to study conducted in Bhutan (67%) [28], Sri Lanka (72.5%) [29], Tanzania (82%) [30] and Uganda (87.8%) [31]. The high prevalence of low fruits and vegetable intake may be due to consumption of more processed food and preference for western diets. Cost barrier can also be considered for low consumption of fruits and vegetables. There was no association between socio-demographic variables and dietary servings of fruits and vegetable intake. The STEPs Survey result of 2013 also revealed no association of insufficient fruits and vegetable intake with socio-demographic characteristics [8]. The prevalence of low fruits and vegetable intake was high (93.9%), therefore, variation in different characteristics was not observed.

It is observed from the study that 92.7% of the respondents consumed salt >5g per day (more than WHO recommendation) which is higher than the study conducted in Nepal (81.6%) [32]. The high prevalence as observed maybe due to overestimation of the consumption of salt by the respondents. Nearly half of the respondents (43.3%) opined that high salt intake doesn’t cause health problem. The study, therefore, suggests culturally tailored dietary modification through reduction in salt intake thereby reducing the risk for hypertension and cardiovascular diseases.

The findings of the current study showed prevalence of low physical activity of 10.2% which is in line with the findings of 2019 Province 3 NCD risk survey (10.3%) [23] and study conducted in Malawi (9.5%) [33]. The prevalence is low in comparison to the study conducted in Iran (42.1%) [34] and South India (49.9%) [35]. Jobs in Nepal are still labor-intensive or there might be over-reporting of physical activity levels. The study suggests balance between occupational and leisure-time physical activity for promotion of better health. There was also no association seen between low physical activity and socio-demographic characteristics. But, although insignificant, the prevalence of physical inactivity was seen high in females than in males which concurs with the study conducted in nine rural HDSSs in five Asian countries: Bangladesh, Indonesia, India, Thailand and Vietnam [36].

### Metabolic risk factors

A high prevalence of overweight and obesity (52.2%) had been observed in the study which is in line with the findings done in semi-urban population of Nepal (52.9%) [37]. The prevalence is low in comparison to the study conducted in Saudi Arabia (54.3%) [38]. However, it is higher in comparison to study conducted in Ethiopia (28.2%) [39], 2019 NCD risk factor survey (24.3%) [7]. Although the prevalence of physical inactivity is low, insufficient fruits and vegetable consumption and shift in intake of highly processed food could be a contributing factor. The findings of the study showed a strong association of overweight or obesity with age, marital status and ethnicity. It was observed that respondents of age groups 30–44 years and 45–59 years were more likely to be overweight/obese than respondents of age group 18–29 years which is similar to the findings of 2013 STEPs survey [8]. A systematic literature review in which fifty–two studies were investigated showed that increasing age was significantly associated with overweight/obesity but that weight seemed to drop again after the age of 50 or 60 [40]. Similar with other studies, the odds of being overweight or obese were more among respondents who are in union in comparison to respondents who are not in union [41, 42]. It is thought that married people are less concerned with their physical appearance than single people who are actively looking for a spouse. Another predictor associated with overweight or obesity is ethnicity and a study conducted in USA have considered race/ethnicity as a factor associated with overweight/obesity [43]. The food preferences of people under different ethnicity influences dietary habit, therefore, a strong association between ethnicity and overweight/obesity might have been observed.

The study has revealed the prevalence of raised blood pressure of 27.8%, which is in line with the findings of 2019 Province 3 NCD risk factor survey (25.2%) [23] and study conducted in Iran (26.6%) [44]. It is higher than the study conducted in Vietnam (18.9%) [45] and Bangladesh (21%) [46]. However, it is lower than the study conducted in Malawi (33%) [33]. Despite being a common issue with a clear diagnosis, hypertension identification and treatment in Nepal is woefully inadequate. Robust policies and programs on NCDs and its risk factors could be one of the approaches to address the problem. Our study demonstrated that older age is a strong factor associated with raised blood pressure, which is in agreement with similar studies conducted in Asian countries [47], Burkina Faso [48], and India [49]. This result serves as a warning to older adults about their increased risk of cardiovascular disease and stroke. The government needs to concentrate more on the issue and take a more holistic approach for handling it. Another finding is that ethnicity is a major determinant of raised blood pressure which is in line with several studies conducted in Asian countries [47]. A study conducted in Nepal concluded that an ethnic variation in the blood pressure distribution in the Nepalese population exists, which might be acting independent of the different life-style factors suggesting more elaborate studies, including longitudinal and migration studies, and probably genetic analyses that can provide more definite power [50].

The prevalence of increased waist hip ratio was 78.4% and the finding was consistent to the study conducted in Singapore (79.8%) [51]. However, it is higher than the study conducted in peri-urban community of Kathmandu (53.9%) [10]. This suggests an expedient need to build strong policies and programs that urges public to have a healthy lifestyle preventing overweight and/or obesity.

### Clustering of behavioral and biological risk factors

Only 3.3% of the study population was free from all the studied NCD risk factors, 65.3% of the respondents had 1–2 risk factors and 31.4% of the respondents with 3–5 risk factors. The findings is pertinent to the study conducted in Kathmandu where 2.0% of the respondents had 0 risk factors, 63.1% had 1–2 risk factors and 34.8% had 3–5 risk factors [18]. The concentration of the risk factors, individually and as clusters increases the risk for cardiovascular disease, chronic respiratory diseases, diabetes and cancer. Future prospective research may be useful in determining the longitudinal progression of NCD risk factors from single to successive concurrence.

Our findings need to be interpreted cautiously in regard to the number of respondents enrolled in the study in view of the number of variables used. The study might have encountered with self-report bias. Also the participants who have lived in another city/country, except the last six months, might have been enrolled. However, the findings of our study are in line with different subnational and national studies carried out in Nepal and different parts of the world.

## Conclusion

The findings of the study revealed high prevalence of behavioral and metabolic risk factors, which varies on different socio-demographic grounds. Therefore, the results of the study can serve as an evidence for policy-makers in fetching out high-risk population to whom health promotion programs aimed at NCDs risk factors reduction can be directed. Also, the findings can provide an overview of the progress of ongoing programs connected with non-communicable diseases.

## Supporting information

S1 Checklist STROBE statement. Checklist of items that should be included in reports of observational studies.
(DOCX)
